# *Pantoea agglomerans* lipopolysaccharide maintains bone density in premenopausal women: a randomized, double-blind, placebo-controlled trial

**DOI:** 10.1002/fsn3.145

**Published:** 2014-07-16

**Authors:** Kazue Nakata, Yoko Nakata, Hiroyuki Inagawa, Takeru Nakamoto, Hiroshi Yoshimura, Gen-Ichiro Soma

**Affiliations:** 1 Department of Nutritional Science, Okayama Prefectural University111 Kuboki, Soja, 719-1197, Japan; 2 Non-profit Organization, Linking Setouchi Innate Network2217-16 Hayashi-cho, Takamatsu, Kagawa, 761-0301, Japan; 3 Department of Integrated and Holistic Immunology, Faculty of Medicine, Kagawa University1750-1 Oaza-ikenobe, Miki-cho, Kida-gun, Kagawa, 761-0793, Japan; 4 Control of Innate Immunity Technology Research Association2217-16, Hayashi-cho, Takamatsu, Kagawa, 761-0301, Japan; 5 Central Park Clinic1-10-16 Ban-cho, Takamatsu, Kagawa, 760-0017, Japan; 6 Nakagawa Hospital2-17-17 Mukaishin-machi, Fukuoka-minami, Fukuoka, 811-1345, Japan; 7 Niigata University of Pharmacy and Applied Life Sciences265-1 Higashijima, Akiha, Niigata, 956-8603, Japan

**Keywords:** Bone density, LPS, osteoporosis, premenopause, soy milk

## Abstract

Lipopolysaccharide from*Pantoea agglomerans* (LPSp) facilitates Ca and P turnover in chicken calvaria and femurs. This study investigated osteoporosis prevention by the oral administration of LPSp in mice and in double-blind clinical tests. Using ovariectomized (OVX) osteoporosis mice model, we investigated the effects of LPSp on the bone density and Ca concentration after ingesting LPSp-containing water for 4 weeks. Oral administration of LPSp tended to suppress the decline in the bone density and the cortical bone thickness in the OVX mice. Moreover, the Ca concentrations were maintained in the OVX-LPSp mice. The effects of LPSp on bone turnover were tested in randomized and double-blind clinical test subjects, who were healthy women aged 40–79 years. The subjects ingested either soy milk without LPSp (control group) or with LPSp (LPSp group) for 3 months. The results showed that the LPSp group on premenopause maintained their bone density compared with the control group pre- and postmenopause. Moreover, these effects were maintained for 2 months postobservation. LPSp maintains bone volume and density in vivo. Thus, a combination of soy milk and LPSp may be useful for osteoporosis prevention.

## Introduction

Osteoporosis is a disease characterized by low bone density and poor bone quality, which causes reduced bone strength and an increased risk of fractures (Faienza et al. 2013). These fractures cause elderly people to become bedridden (Hagino et al. 2006). Bedridden patients may also experience dementia and pneumonia, which can increase the costs of medical care and decrease the quality of life (QOL). Thus, osteoporosis prevention is very important for reducing the medical care costs and increasing the QOL because treatments are ineffective after fractures.

The bone mass is mainly regulated by osteoblasts (bone formation) and osteoclasts (bone resorption) (Lewiecki 2011). Bone is the major store of calcium (Ca) and a key regulatory organ for Ca homeostasis. Osteoclasts are sensitive to partial defects in Ca maintenance, express Ca-binding proteins, and support significant Ca transport (Blair et al. 2011). Thus, osteoblasts and osteoclasts play important roles in regulating the bone mass and Ca metabolism. The prevention and treatment of osteoporosis requires the intake of Ca (the major bone component) and vitamin D (a promoter of Ca absorption) (Garriguet 2011). However, the Ca intake is usually inadequate in Japanese men and women because of their food preferences.

Furthermore, estrogen enhances bone formation through the repression of osteoclastogenic cytokine production from immune cells, increasing osteoblast proliferation, decreasing osteoblast and osteocyte apoptosis, and induction of osteoclast apoptosis (Krum 2011). Thus, the risk of osteoporosis is increased in postmenopausal women because of their reduced estrogen production. Soy isoflavone, which has similar but weak effects to estrogen, is known to regulate bone metabolism (Yamaguchi 2006). It has been reported that osteoporosis may be prevented by the ingestion of Ca and soy isoflavone (Wong et al. 2009; Alekel et al. 2010; Verbrugge et al. 2012), but they are insufficient to prevent osteoporosis. Thus, the development of new food ingredients that prevent osteoporosis is very important. Therefore, if a safe food could be developed to regulate normal bone metabolism based on mechanisms other than isoflavone and Ca, it may be utilized to prevent osteoporosis with their combinations.

Macrophages play a role in the maintenance of body homeostasis by preventing infections, eliminating waste products, healing wounds and fractures, and regulating metabolism such as bone metabolism (Stefater et al. 2011). Thus, the decline in the structure of bone with advancing age may be prevented by activated macrophages. We identified macrophage-activating substances in a water extract of wheat flour (Tsukioka et al. 1997). This activating substance was the lipopolysaccharide (LPS) produced by the Gram-negative symbiotic bacterium,*Pantoea agglomerans*.*Pantoea agglomerans* is found in many food plants (Asis and Adachi 2004; Miao et al. 2008; Quecine et al. 2012) and is required for the fermentation of rye sourdough (Kariluoto et al. 2006). In Europe, live*P. agglomerans* is also used as a biocontrol agent to prevent the fungal spoilage of fruit (Kamber et al. 2012) and it has been confirmed as a safe substance for oral consumption. The effects of*P. agglomerans* LPS (LPSp) have been reported in animal and human clinical trials, and it has been shown to improve diabetes, dyslipidemia (Iguchi et al. 1992; Okutomi et al. 1992a; Nakata et al. 2011), and atopic dermatitis, as well as preventing infections (Nakamoto et al. 2007) and reducing pain (Okutomi et al. 1992b,c).

Previously, LPSp was shown to have the potential to promote bone turnover in chick embryo ex vivo. It has been reported that LPSp promoted bone formation and bone absorption because the total Ca and phosphorus (P) concentrations in bone were increased by LPSp (Kawashima et al. 1992). Thus, if the bone metabolism can be maintained and/or the bone mass increased via the oral intake of LPSp, it may be possible to utilize LPSp as a novel mechanism for osteoporosis prevention. The mechanism of action of LPSp is probably different from that of isoflavone, because isoflavones regulate bone metabolism by binding to estrogen receptors similar to estrogen (Yamaguchi 2006). Therefore, there may be a synergistic effect between LPSp and isoflavone.

In this study, we focused on LPSp and its combination with isoflavone as a possible safe food to regulate normal bone metabolism, which may be utilized to prevent osteoporosis with their combinations. On the basis of a previous experiment with chick embryo ex vivo, we first confirmed the effects of orally administered LPSp in mice. We found a possible preventive effect of LPSp on osteoporosis by feeding osteoporosis model mice with LPSp. On the basis of this result, we also investigated a combination of soy milk with LPSp (the fermented flour extract) to determine whether it improved the bone concentration and metabolism in women aged over 40 years in a randomized, double-blind trial.

## Materials and Methods

### Study products

The fermented flour extract was produced by MACROPHI Inc. (Kagawa, Japan) and contained 0.1 g of LPSp per 10 g. The products used in the clinical study are listed in Table1. The fermented flour extract contained 60 mg of the test products per 12.5 g. Both the control and the test products contained 30 mg of the soy isoflavone extract and the total amount of isoflavone was 13.5 mg.

**Table 1:** Composition of experimental samples (mg/12.5 g).

	Control	LPSp
Soymilk powder	5000	5000
Germinated soybean powder	1250	1250
Dextrin	4446	4446
Oil powder	25.0	25.0
Soy isoflavone extract	30.0	30.0
Shell calcium^1^	1621	1621
The fermented flour extract (LPSp)^2^	0	60.0
Vitamin C	45.0	45.0
Vitamin B complex^3^	2.25	2.25
Vitamin D	0.5	0.5
Folic acid	20.0	20.0

Total isoflavone content was ˜13.5 mg. Vitamin C is added for antioxidation and vitamin B complex and folic acid are added for enhancing of metabolism.

1 Calcium contents: 600 mg.

2 LPSp contents: 0.6 mg.

3 Vitamin B complex contents (12.5 g): B1, 0.147 mg; B2, 0.743 mg; B6, 0.383 mg; nicotinamide, 0.765 mg; calcium pantothenate, 0.165 mg.

### Animals and experimental protocol

Ten-week-old female C57BL/6J wild-type mice were obtained from CLEA Japan Inc. (Tokyo, Japan). The mice were quarantined in local vivarium conditions (24°C and 12/12 h light/dark cycle) for 1 week. The animal diets are described in Table2. The mice were randomized by body weight into the following three groups: three mice were sham operated and ingested distilled water (DW) (SHAM-DW mice), five mice were ovariectomize (OVX) and ingested DW (OVX + DW mice), and five mice were both OVX and ingested LPSp (OVX + LPSp mice). LPSp dose setting was based on previous studies (Nakata et al. 2011), and the mice ingested LPSp at 0.05 *μ*g/mL day^−1^. The sham-operated and OVX mice were allowed free access to DW and fermented flour extract for 4 weeks. After 4 weeks, these mice were euthanized and their femurs were collected. The analyses of the bone density, Ca concentration, and cortical bone thickness in the femur were performed by ELK Corporation (Osaka, Japan). The experimental procedures were reviewed and approved by Kagawa University (Permission No. 139).

**Table 2:** Composition of animal diets per 100 g of product.

Nutrition	
Moisture (g)	8.9
Crude protein (g)	24.9
Crude fat (g)	4.6
Crude fiber (g)	4.1
Minerals	
Calcium (g)	1.06
Phosphorus (g)	0.99
Vitamins	
Vitamin C (mg)	25
Vitamin B1 (mg)	2.0
Vitamin B2 (mg)	1.3
Vitamin B6 (mg)	1.4
Vitamin B12 (*μ*g)	5.6
Vitamin D3 (IU)	205
Folic acid (mg)	0.2

### Subjects and Methods

The study protocol was approved (Permission Nos. H21-1 and H22-1) by the Ethics Committee of the Non-profit Organization-Linking Setouchi Innate Immune Network (NPO-LSIN), and it was performed in accordance with the principles of the Declaration of Helsinki. All examinations were managed by a doctor at Central Park Clinic (Takamatsu, Kagawa) which is a NPO-LSIN coalition medical institution.

The morbidity related to osteoporosis increases in menopausal women aged >40 years; therefore, the subjects were women aged 40–80 years. The exclusion criteria were as follows: (i) taking vitamin D supplements; (ii) taking functional foods and medicines, including soy isoflavone, within 1 week of examination; (iii) patients with hypercalcemia, osteoporosis, and cancer; and (iv) women who were judged to be unsuitable by a responsible doctor.

The subjects were previous or new participants in NPO-LSIN. The subjects signed a consent form after being provided with oral and written descriptions of the study.

### Study design and intake methods

The study was double-blinded and randomized using envelopes. The study comprised two periods: the product intake period lasting 3 months from baseline and the subsequent 2 months were used for postobservation. The subjects maintained their lifestyle, such as diet, physical activity, and smoking, at levels similar to the baseline and their product intake, physical condition, and menstruation were recorded throughout the study period. At breakfast, each subject ingested the product, which was dissolved in ˜100 mL cold or hot water.

### Assessments

Medical assessments were performed three times in the Central Park Clinic, as follows: at baseline, at the end of the 3-months intake period (+3 months), and 2 months after the end of postobservation (−2 months). Blood samples were collected after an overnight fast. Body height, weight, fat rate, and bone density (dual-energy X-ray absorptiometry [DXA], Aloka Dichroma Scan DCS-600EX-II) were measured. Body mass index (BMI) was calculated as kg/m^2^. Bone metabolic markers were measured based on bone-specific alkaline phosphatase (BAP), cross-linked N-telopeptides of type 1 collagen (NTx), and serum Ca levels. BAP and NTx were analyzed by BML Inc. (Tokyo, Japan). The serum Ca level was analyzed by Shikoku Chuken INC (Kagawa, Japan).

### Statistical analyses

The data obtained from the animal experiment and clinical tests were expressed as the mean ± SE.*P *<* *0.05 were considered significant. The statistical analyses were performed using Excel Statistics 2008 (Microsoft, Redmond, WA). In the animal experiment, the differences between the SHAM-DW and SHAM-LPSp groups and OVX-DW or OVX-LPSp groups were analyzed using Student's*t*-tests. In the clinical trial, the differences between the control and LPSp groups were compared using a two-way analysis of variance.*P *<* *0.05 were considered significant.

## Results

### The confirmation of LPSp effect in the osteoporosis model mice as a pilot study

**Figure 1:**
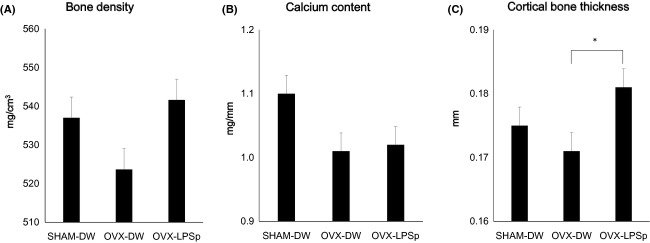
Change in (A) the bone density, (B) Ca concentration, and (C) cortical bone thickness by ingested LPSp in osteoporosis model mice. The OVX-LPSp mice ingested LPSp (0.05 *μ*g/mL^−1^ day^−1^) for 4 weeks. The bone density, Ca concentration, and the cortical bone thickness were analyzed in the femur. Displayed are mean + SE. *P <* *0.05.

We confirmed the effects of ingesting a fermented flour extract that contained LPSp using the OVX mice. Sham-operated or OVX mice had free access to drinking water or the fermented flour extract (LPSp included 0.05 *μ*g/mL) for 4 weeks. We measured their bone density, the Ca concentration, and the cortical bone thickness in the femur. The bone densities of each group were as follows: SHAM-DW mice, 537.0 ± 2.5 mg/cm^3^; OVX-DW mice, 523.7 ± 6.1 mg/cm^3^; and OVX-LPSp mice, 541.6 ± 10.1 mg/cm^3^. The OVX-DW mice had lower bone densities compared with the SHAM-DW mice, while the OVX-LPSp mice had higher densities than the OVX-DW mice. The OVX-LPSp mice were similar to the SHAM-DW (Fig.1A). The Ca concentrations of each group were as follows: SHAM-DW mice, 1.10 ± 0.04 mg/mm; OVX-DW mice, 1.01 ± 0.02 mg/mm; and OVX-LPSp mice, 1.02 ± 0.04 mg/mm. The OVX-DW and the OVX-LPSp mice had lower concentrations than the SHAM-DW mice (Fig.1B). The cortical bone thickness of each group was as follows: SHAM-DW mice, 0.175 ± 0.005 mm; the OVX-DW mice, 0.171 ± 0.002 mm; and the OVX-LPSp mice, 0.181 ± 0.004 mm. The OVX-DW mice had a lower mean bone thickness compared with the SHAM-DW and OVX-LPSp mice. The OVX-LPSp mice were similar to the SHAM-DW mice (Fig.1C). The bone density of OVX-LPSp mice tended to increase (*P* = 0.15) compared to that of OVX-DW. There were no significant differences between the OVX-DW and the OVX-LPSp mice in Ca concentration, while the cortical bone thickness in the OVX-LPSp mice significantly increased (*P* < 0.05). Thus, total Ca amount was suggested increasing in the OVX-LPSp mice. These results suggest that LPSp helps to prevent osteoporosis.

### Baseline characteristics of the study population

We investigated the potential application of LPSp for the osteoporosis prevention using soy milk with or without the fermented flour extract. The study subjects were 52 healthy women aged 40–79 years. Four of 52 women dropped out for the following reasons: the day of the clinical check was unsuitable and they were anxious about disturbing their menstruation during the intake period. The characteristics of the subjects before the clinical trial commenced are shown in Table3. Soy milk without the fermented flour extract group (control) was provided to 22 subjects and soy milk with the fermented flour extract group (the LPSp group) was provided to 26 subjects. The control group and LPSp group contained 11 and 10 premenopausal women, respectively. The age of each group were as follows: the premenopause-control (the pre-control) group 44.9 ± 3.8 years, the postmenopause-control (the post-control) group 58.0 ± 4.9 years, the pre-LPSp group 45.4 ± 2.8 years, and the post-LPSp group 58.0 ± 4.9 years. The body weight, fat, BMI, and bone density of the subjects were similar in each group.

**Table 3:** Baseline characteristics of the study population.

	Control	LPSp
Number	22	26
Premenopause	11	10
Postmenopause	11	16
Age	52.8 ± 8.6	52.3 ± 7.6
Premenopause	44.9 ± 3.8	45.4 ± 2.8
Postmenopause	58.0 ± 4.9	58.0 ± 4.9
Body weight (kg)	53.0 ± 8.4	54.4 ± 10.5
Premenopause	52.1 ± 11.5	50.8 ± 4.9
Postmenopause	53.6 ± 5.6	54.0 ± 6.0
Body fat (%)	27.1 ± 6.8	28.2 ± 6.3
Premenopause	27.4 ± 9.0	28.0 ± 4.4
Postmenopause	26.9 ± 5.0	26.5 ± 5.0
BMI (kg/m^2^)	22.1 ± 3.3	22.6 ± 3.7
Premenopause	22.1 ± 4.4	21.4 ± 2.7
Postmenopause	22.1 ± 2.4	22.7 ± 2.5

All numerical values are presented as mean ± SD.

### Changes in the bone density, bone alkaline phosphatase, NTx, and serum Ca

In this study, subjects who forgot to take the products are not represented in the intake period. The bone density of normal adults is about 1.0 g/cm^2^. The mean bone density of the control group at baseline, +3 months, and −2 months was 0.62 ± 0.10, 0.61 ± 0.10, and 0.61 ± 0.10 g/cm^2^, respectively (Table4). In the LPSp group, the mean bone densities at baseline, +3 months, and −2 months were 0.60 ± 0.12, 0.59 ± 0.12, and 0.59 ± 0.12 g/cm^2^, respectively. There were no significant differences among the treatment groups, thus we compared the changes relative to the baseline. The results showed that oral administration of LPSp significantly prevented the decreasing bone density than that of the control group and this effect continued until −2 months: the control group at +3 months, 97.7 ± 3.4% and at −2 months, 97.4 ± 4.3%; the LPSp group at +3 months, 98.6 ± 2.1% and at −2 months, 98.8 ± 2.6% (Table5). We also compared the pre- and postmenopause groups, and the pre-LPSp group maintained their mean bone density at +3 months, 99.1 ± 1.9%. However, the bone density in the post-LPSp group at +3 months was lower than the 0 month, 98.1 ± 2.3%.

**Table 4:** Changes in body composition and bone metabolic markers values in subjects after LPSp ingestion.

	Control group (isoflavone)			LPSp group (isoflavone + LPSp)		
	0 month	+3 months	−2 months	0 month	+3 months	−2 months
Height (cm)	155.0 ± 10.9	–	–	155.0 ± 4.8	–	–
Body weight (kg)	53.0 ± 8.4	53.9 ± 8.4	54.0 ± 8.6	54.4 ± 10.5	55.2 ± 10.9	52.8 ± 5.6
Premenopause	52.1 ± 11.5	53.4 ± 11.4^1^,**	53.4 ± 11.6^2^,**	50.8 ± 4.9^1^,*	51.4 ± 4.8	51.0 ± 5.1
Postmenopause	53.6 ± 5.6	54.4 ± 5.8^1^,*	54.5 ± 5.8^2^,**	54.0 ± 6.0^1^,*	54.8 ± 5.8	54.9 ± 5.6
Body fat (%)	27.1 ± 6.8	30.2 ± 6.6	30.0 ± 7.0	28.2 ± 6.3	27.6 ± 5.1	28.3 ± 5.1
Premenopause	27.4 ± 9.0	30.0 ± 8.3	30.1 ± 8.7	26.5 ± 5.0	27.0 ± 4.7	26.5 ± 4.8
Postmenopause	26.9 ± 5.0	30.4 ± 5.1	30.0 ± 5.8	28.0 ± 4.4	28.1 ± 5.7	30.8 ± 5.0
BMI (kg/m^2^)	22.1 ± 3.3	22.5 ± 3.3	22.5 ± 3.4	22.6 ± 3.7	22.9 ± 3.7	22.2 ± 2.6
Premenopause	22.1 ± 4.4	22.7 ± 4.3^1^,**	22.7 ± 4.5^2^,**	21.4 ± 2.7	21.6 ± 2.5	21.4 ± 2.7
Postmenopause	22.1 ± 2.4	22.3 ± 2.5	22.3 ± 2.4^2^,*	22.7 ± 2.5	23.0 ± 2.5^1^,*	22.9 ± 2.6
Bone density (g/cm^3^)	0.62 ± 0.1	0.61 ± 0.1	0.61 ± 0.1	0.60 ± 0.12	0.59 ± 0.12	0.59 ± 0.12
Premenopause	0.69 ± 0.1	0.67 ± 0.1	0.67 ± 0.1	0.70 ± 0.1	0.69 ± 0.1	0.69 ± 0.1
Postmenopause	0.57 ± 0.1	0.56 ± 0.1	0.56 ± 0.1	0.51 ± 0.1	0.50 ± 0.1	0.51 ± 0.1
BAP (*μ*g/L)	13.4 ± 4.2	13.6 ± 4.6	14.7 ± 4.9	15.3 ± 6.1	16.4 ± 7.7	16.7 ± 7.7
Premenopause	10.2 ± 2.4	10.8 ± 3.4	11.3 ± 3.1	10.8 ± 2.5	11.2 ± 2.8	11.3 ± 2.7
Postmenopause	15.8 ± 3.6	15.7 ± 4.4	17.3 ± 4.3	19.1 ± 5.5	20.7 ± 7.9	21.2 ± 7.6
NTx (NMBCE/L)	17.2 ± 6.5	17.9 ± 6.9	17.5 ± 6.2	18.45 ± 7.3	18.6 ± 7.7	18.4 ± 7.3
Premenopause	12.2 ± 2.8	12.3 ± 1.7	12.6 ± 2.6	15.8 ± 8.2	16.5 ± 9.5	16.2 ± 8.3
Postmenopause	20.9 ± 6.0	21.9 ± 6.3	21.1 ± 5.5	20.6 ± 6.1	20.4 ± 5.6	20.3 ± 6.0
Ca (mg/dL)	9.35 ± 0.32	9.46 ± 0.37	9.37 ± 0.33	9.41 ± 0.28	9.54 ± 0.32	9.42 ± 0.36
Premenopause	9.13 ± 0.26	9.20 ± 0.27	9.16 ± 0.21	9.33 ± 0.30	9.44 ± 0.34	9.25 ± 0.25
Postmenopause	9.51 ± 0.26	9.65 ± 0.32	9.52 ± 0.33	9.48 ± 0.26	9.63 ± 0.28	9.57 ± 0.38

The LPSp group ingested LPSp (0.6 mg/day). Subjects ingested test product for 3 months period, and did not ingest after 2 months. Medical assessments were held three times: 0 month is baseline, +3 months is products intake period 3 months from baseline, and −2 months is products not intake period after 2 months. All numerical values are presented as mean ± SD.

* *P *<* *0.05,

** *P *<* *0.01.

1 0 month versus +3 months.

2 0 month versus −2 months.

**Table 5:** Changes of ratio of body composition and bone metabolic markers in subjects after LPSp ingestion.

	Control group (isoflavone)			LPSp group (isoflavone + LPSp)		
	0 month	+3 months	−2 months	0 month	+3 months	−2 months
Body weight	100	101.9 ± 2.1	102.1 ± 2.1	100	101.5 ± 1.9	100.5 ± 2.2
Premenopause		102.7 ± 1.7^1^,**	102.8 ± 2.4^2^,**		101.3 ± 1.7^1^,*	100.4 ± 1.5
Postmenopause		101.4 ± 2.2^1^,*	101.6 ± 1.8^2^,*		101.5 ± 2.1^1^,*	100.7 ± 2.9
Body fat	100	109.0 ± 6.6	108.9 ± 6.5	100	106.1 ± 5.6	106.2 ± 6.8
Premenopause		112.2 ± 6.9^1^,**	112.5 ± 8.0^2^,**		105.1 ± 5.4^1^,*	103.4 ± 6.4
Postmenopause		105.7 ± 4.4^1^,**	106.1 ± 3.3^2^,**		106.5 ± 5.3^1^,**	109.4 ± 6.2^2^,**
BMI	100	101.7 ± 2.0	101.8 ± 2.2	100	101.3 ± 1.8	100.4 ± 2.1
Premenopause		102.5 ± 1.8^1^,**	102.6 ± 2.4^2^,**		101.0 ± 1.7	100.1 ± 1.5
Postmenopause		101.2 ± 2.1^1^,*	101.1 ± 1.8^2^,*		101.4 ± 2.0^1^,*	100.7 ± 2.5
Bone density	100	97.7 ± 3.4^1^,**	97.4 ± 4.3^3^,**	100	98.6 ± 2.1^1^,**	98.8 ± 2.6^3^,*
Premenopause		97.8 ± 3.5^1^,**	96.9 ± 4.5^3^,**		99.1 ± 1.9	99.1 ± 2.5
Postmenopause		97.7 ± 3.3^1^,**	97.8 ± 4.3^3^,**		98.1 ± 2.3^1^,**	98.6 ± 2.8
BAP	100	102.0 ± 15.2	110.0 ± 13.8^2^,**,^3^,**	100	106.3 ± 17.0^1^,*	108.1 ± 18.2^3^,**
Premenopause		107.9 ± 17.7	110.9 ± 11.0^3^,**		105.0 ± 17.4	106.2 ± 19.2
Postmenopause		98.5 ± 12.6	109.4 ± 15.7^2^,**,^3^,**		107.4 ± 17.5^1^,*	109.7 ± 18.1^3^,**
NTx	100	104.7 ± 13.1	104.5 ± 18.7	100	101.9 ± 18.3	101.0 ± 17.0
Premenopause		103.5 ± 16.0	106.4 ± 24.0		104.1 ± 19.4	103.0 ± 15.1
Postmenopause		105.5 ± 11.0	103.0 ± 14.3		100.0 ± 18.1	99.4 ± 19.0
Ca	100	101.3 ± 3.0^1^,**	100.2 ± 2.8^3^,*	100	101.4 ± 2.8^1^,**	100.3 ± 2.8^3^,**
Premenopause		100.8 ± 3.2	100.4 ± 2.5		101.2 ± 3.2	99.2 ± 2.6
Postmenopause		101.6 ± 2.9	100.2 ± 3.1		101.6 ± 2.7	101.0 ± 2.9

The LPSp group ingested LPSp (0.6 mg/day). Subjects ingested test product for 3 months period, and did not ingest after 2 months. Medical assessments were held three times; 0 m is baseline, +3 m is products intake period 3 months from baseline, and −2 months is products not intake period after 2 months. These data compared the changes relative to the baseline. All numerical values are presented as mean ± SD.

* *P *<* *0.05,

** *P *<* *0.01.

1 0 month versus +3 months.

2 0 month versus −2 months.

3 +3 months versus −2 months.

Bone metabolism markers include BAP, which is a marker of osteogenesis, and NTx, which is a marker of bone resorption. BAP is an enzyme that is present in large amounts in osteoblast, and it reflects the total number of osteoblasts and the potential osteogenesis function. A reduction in the BAP level suggests possible bone loss. The normal premenopause BAP value is 2.9–14.5 *μ*g/L and the postmenopause value is 3.8–22.9 *μ*g/L (Table4). The results showed that the values in both groups were normal during the experimental period. The changes in the control group were: +3 months, 102.0 ± 15.2%; −2 months, 110.0 ± 13.8%, and the changes in the LPSp group were: +3 months, 106.3 ± 17.0%; −2 months, 108.1 ± 18.2% (Table5). The BAP levels of both groups tended to increase, although they did not change significantly. A comparison of the levels in the pre- and postmenopausal groups showed that the post-LPSp group at +3 months had significant increased BAP levels, 107.4 ± 17.5%, which were maintained at −2 months, 109.7 ± 18.1%. NTx is the degradation product of cross-linked N-telopeptides from type 1 collagen, which is the main protein substrate in bone. NTx is known to be associated with the degradation of bone. The normal premenopause value is 7.5–16.5 NMBCE/L and the postmenopause value is 10.7–24.0 NMBCE/L. If the NTx level exceeds the mean by 1.0 SD compared with normal premenopausal women, a high risk of bone loss is indicated. The baseline values in each group were higher than the normal premenopausal value: the control group, 17.2 ± 7.3 NMBCE/L, and the LPSp group, 18.5 ± 6.5 NMBCE/L (Table4). The changes in NTx in the control group were: +3 months, 104.7 ± 13.1%; −2 months, 104.5 ± 18.7% and in the LPSp group were: +3 months, 101.9 ± 18.3%; −2 months, 101.0 ± 17.0% (Table5). The NTx level of the pre-control, post-control, and pre-LPSp groups increased slightly, whereas the post-LPSp group tended to decrease, but there were no significant differences between the groups. We also measured the serum Ca level, which is related to bone metabolism, and the results for the control group were: baseline, 9.4 ± 0.3 mg/dL; +3 months, 9.5 ± 0.3 mg/dL; −2 months, 9.4 ± 0.4 mg/dL and for the LPSp group were: baseline, 9.4 ± 0.3 mg/dL; +3 months, 9.5 ± 0.4 mg/dL; −2 months, 9.4 ± 0.3 mg/dL (Table4). There was a significant increase from the baseline level to +3 months in each group (*P *<* *0.01). However, it returned to the same level as the baseline when the treatment was discontinued. There was no significant difference between the control and LPSp group. The same results were obtained in the pre- and postmenopause groups.

### The effects of menstruation and physical condition

The subjects who did not experience any changes in their menstrual cycle during the intake period comprised four women in the control group and eight women in the LPSp group.

Changes in menstrual pain were experienced by six women in the control group and ten women in the LPSp group, although none experienced severe menstrual pain. The physical condition of the subjects suggested that their bowel movements were affected, including regular defecation or diarrhea.

## Discussion

In this study, the possible utilization of LPSp for osteoporosis prevention was confirmed by testing the effects of ingesting a*P. agglomerans*-fermented flour extract in osteoporosis model mice as performed a for human pilot study. We investigated whether LPSp improved bone metabolism in human subjects using a randomized and double-blind clinical study. We investigated whether LPSp improved bone metabolism in human subjects using a randomized and double-blind clinical study. We confirmed the adequate LPSp dose (10–20 *μ*g kg^−1^ day^−1^ by oral ingestion) from various kinds of animal species experiments with shellfish, fish, chick, dog, mouse, and human against various diseases protection such as infectious diseases, allergy diseases, and cancer (Kohchi et al. 2006; Kadowaki et al. 2013). In general, the average weight of mice is ˜25 g, and the average daily amount drunk is ˜5 mL. The ingestion amount of LPSp in the mice experiments was accordingly set at 10 *μ*g kg^−1^ day^−1^. Similarly, in composing the products for human experiments, we made test products containing 60 *μ*g of LPSp because we postulated an average weight of 60 kg (10 *μ*g kg^−1^ day^−1^ of LPSp). In fact, the amount of LPSp in this study was 11 *μ*g kg^−1^ day^−1^ because the weights of subjects were 54.4 ± 10.5 kg for the LPSp group. These doses implied the equality of a previously determined amount.

It has been reported that LPSp activated the bone metabolism directly in chicken parietal bone and femur culture ex vivo experiments (Kawashima et al. 1992). The Ca release from the isolated chicken fetal parietal bone was stimulated to a similar level with parathyroid hormone (PTH). Moreover, LPSp has been shown to increase bone formation, whereas PTH has not. In the osteoporosis model mice, LPSp ingestion was effective to prevent osteoporosis in OVX mice. Although the OVX-LPSp mice had no significant difference in the Ca concentration (Fig.1B), the cortical bone thickness was significantly increased (*P* = 0.05) (Fig.1C). Therefore, LPSp ingestion inferred that the total Ca was increased. Moreover, the bone density of OVX-LPSp mice tended to increase (*P* = 0.15) compared to that of OVX-DW (Fig.1A). This result does not show statistically significant difference because this study exhibited large variance. We used a small number of groups because of a pilot study. In addition, we used different number of animals because the OVX group assigned a large number of animals to minimize the effect of large variance for the OVX operation. Although we did not investigate an LPSp + isoflavone group, our results suggested that LPSp has the potential to maintain bone turnover with low estrogen, thus confirming the specific effect of LPS oral ingestion on bone.

In the clinical study, the bone density was maintained in the LPSp group, and this effect remained for 2 months after the treatment was discontinued (Table5). The subjects were divided into pre- and postmenopause groups, and the bone density maintenance was significantly better in premenopausal women (Table5). However, the levels of the bone metabolic markers in the present clinical study did not reflect this effect (Table5). The bone density declined in the post-LPSp group, while the BAP levels tended to increase in the early stage (Table5). The BAP level is correlated with the total number of osteoblasts and the potential osteogenesis function. In general, bone metabolism is studied over the course of 6 months or several years because bone turnover requires several months (Arjmandi et al. 2005; Brink et al. 2008; Wong et al. 2009). The effects of soy milk in Asian people for biological improvement, such as an increase in the vaginal intermediate type cells and tendency for alkaline phosphatase, were shown in 3 months of ingestion. However, serum Ca and bone mineral density were unchanged (Uesugi et al. 2003). Thus, the observational period used in the present study to detect changes in some biomarkers are possible, but bone markers (BAP, NTx, and Ca) may have been insufficient with a treatment period of only 3 months.

Although the bone density maintenance effect was observed in pre-LPSp group but not in post-LPSp group (Table5), it may have been affected by estrogen. Estrogen induces the differentiation of osteoblasts and enhances bone formation (Beck and Hansen 2004). However, estrogen also suppresses bone resorption by directly inducing osteoclast apoptosis (Kameda et al. 1997). Estrogen indirectly inhibits the induction of interleukin (IL)-1*β*, IL-6, IL-7, and tumor necrosis factor-*α* secretion from osteoblasts and bone marrow stromal cells (Weitzmann and Pacifici 2006; D'Amelio et al. 2011), while it enhances Ca absorption from the digestive tract. Postmenopausal women experience increased bone resorption and decreased Ca absorption by the gut; therefore, it may be impossible to maintain their bone density with a Ca deficit even if their osteoblasts are enhanced by LPSp. To clarify this mechanism, the serum estrogen levels will be measured in future to investigate the possible synergistic effects of estrogen and LPSp.

The soy milk used in this study contained 30 mg of soy isoflavone extract. This amount was less than 50% of the recommended maximum daily intake value (70–75 mg/day) defined by the Food Safety Commission in Japan (Branca 2003; Coxam 2008). This amount of isoflavone in the soy milk is inadequate, but it may have compensated for the decreased estrogen levels of the middle-aged and elderly women in this study.

These results suggest that LPSp facilitates the development of an osteoporosis prevention strategy with some combinations because LPSp with soy milk was effective in maintaining the bone density of premenopausal women.
