# *Pantoea agglomerans* lipopolysaccharide maintains bone density in premenopausal women: a randomized, double‐blind, placebo‐controlled trial

**DOI:** 10.1002/fsn3.327

**Published:** 2016-01-08

In Nakata et al. (2014), Table 1 was published with errors. The product names “Vitamin D” and “Folic acid”, and its legends have been amended.

Corrected Table 1 is shown below.

**Table 1:** Composition of experimental samples (mg/12.5 g)

	Control	LPSp
Soymilk powder	5000	5000
Germinated soybean powder	1250	1250
Dextrin	4446	4446
Oil powder	25.0	25.0
Soy isoflavone extract	30.0	30.0
Shell calcium^a^	1621	1621
The fermented flour extract (LPSp)^b^	0	60.0
Vitamin C	45.0	45.0
Vitamin B complex^c^	2.25	2.25
Vitamin D extract^d^	0.5	0.5
Folic acid extract^e^	20.0	20.0

Total isoflavone contents: approximately 13.5 mg.

a Calcium contents: 600 mg.

b LPSp contents: 0.6 mg.

c Vitamin B complex contents (12.5 g): B1, 0.147 mg; B2, 0.743 mg; B6, 0.383 mg; Nicotinamide, 0.765 mg; Calcium pantothenate, 0.165 mg. Vitamin C is added for antioxidation, Vitamin B complex and Folic acid are added for enhancing of metabolism.

d Vitamin D contents: 0.0025 mg.

e Folic acid contents: 0.2 mg.

We apologize for these errors.

## References

[fsn3327-bib-0001] Nakata, K. , Y. Nakata , H. Inagawa , T. Nakamoto , H. Yoshimura , and G.‐I. Soma . 2014 *Pantoea agglomerans* lipopolysaccharide maintains bone density in premenopausal women: a randomized, double‐blind, placebo‐controlled trial. Food Sci. Nutr. 2:638–646. 25493180 10.1002/fsn3.145 PMC4256567

